# Characterization of Ionic Liquid Lignins Isolated from Spruce Wood with 1-Butyl-3-methylimidazolium Acetate and Methyl Sulfate and Their Binary Mixtures with DMSO

**DOI:** 10.3390/molecules25112479

**Published:** 2020-05-27

**Authors:** Artyom V. Belesov, Anton V. Ladesov, Ilya I. Pikovskoi, Anna V. Faleva, Dmitry S. Kosyakov

**Affiliations:** Core Facility Center ‘Arktika’, Northern (Arctic) Federal University, Arkhangelsk 163002, Russia; a.belesov@narfu.ru (A.V.B.); a.ladesov@narfu.ru (A.V.L.); i.pikovskoj@narfu.ru (I.I.P.); a.bezumova@narfu.ru (A.V.F.)

**Keywords:** lignin, ionic liquids, 1-butyl-3-methylimidazolium, wood fractionation

## Abstract

Ionic liquids (ILs) based on 1-butyl-3-methylimidazolium (bmim) cation have proved to be promising solvents for the fractionation of plant biomass with the production of cellulose and lignin. This study deals with the characterization of lignins isolated from coniferous (spruce) wood using [bmim]OAc and [bmim]MeSO_4_ ionic liquids and their binary mixtures with DMSO (80:20). Molecular weight distributions, functional composition, and structural features of IL lignins were studied by size-exclusion chromatography, NMR spectroscopy (1D and 2D) and atmospheric pressure photoionization high-resolution mass spectrometry. It was shown that the interaction of ILs with lignin leads to significant chemical changes in the biopolymer; a decrease in the degree of polymerization and in the content of free phenolic hydroxyl groups due to alkylation, the disappearance (in the case of [bmim]OAc) of carbonyl groups and a significant destruction of β-*O*-4 bonds. The chemical reactions between lignin and 1-butyl-3-methylidazolium cation with covalent binding of ionic liquids or products of their decomposition is evidenced by the presence of a large number of nitrogen-containing oligomers in IL lignins.

## 1. Introduction

One of the most promising areas of chemical processing of renewable plant feedstock compliant with green chemistry principles is the fractionation of lignocellulosic biomass using ionic liquids (ILs). It is known that ILs based on 1-ethyl- and 1-butyl-3-methylimidazolium cations (emim and bmim, respectively) [1,2,3] have an exceptionally high dissolving power in relation to lignin and polysaccharides and a unique ability to fully dissolve wood and other types of lignocellulosic raw materials [4]. At the same time, ILs are characterized by such valuable properties as non-flammability, low vapor pressure, thermal stability and the possibility of regeneration and reuse in the technological processes. One of the most important advantages of ionic liquids is the possibility of targeted tuning of their properties by selection of anion, the nature of which can significantly affect the solvating power and reactivity of ILs [5,6,7]. In order to reduce the cost and viscosity of ionic liquids, molecular solvents, such as dimethyl sulfoxide, are used as additives, which at certain concentration ranges do not radically change the structure and properties of ILs [8]. Polysaccharide (cellulose and hemicellulose) and lignin fractions can be extracted from the obtained solutions of plant biomass by fractional sedimentation with water and some other solvents [9].

While polysaccharides can be successfully used to produce various materials and as a feedstock for further chemical processing (e.g., conversion into monosaccharides and furfural), effective methods of valorization of the IL lignins have not yet been developed. This is due to both the complexity of the biopolymer structure, variety of its aromatic units and functional groups, and a lack of knowledge on IL lignins, the studies of which are only beginning to show interest in recent years. The information currently available in the literature on this topic is still quite fragmentary.

It is known that ILs in relation to lignin can act not only as highly efficient solvents, but also as reagents that can actively interact with the biopolymer, causing its chemical transformation. The IL treatment of lignin at higher temperatures mainly leads to degradation of the most labile β-*O*-4 ether bonds [10,11]. At the same time, condensation processes are also possible, for example, with the formation of α-5 and β-5 carbon–carbon bonds [12,13]. The synergy of these factors can lead to both a decrease and increase in the molecular weight of the obtained IL lignins during their extraction from plant biomass. In addition to depolymerization/condensation processes, other chemical transformations can also occur under the influence of ILs, which largely depend on properties of IL anion. Lignin isolated using emim acesulfamate [14] had an increased content of carbonyl groups, and both nitrogen and sulfur were found in its elemental composition. This suggested the possibility of chemical interaction of acesulfamate anion with lignin [15]. Similarly, the formation of carbonyl and quinone structures in addition to demethoxylation of lignin was observed under the treatment with 1-butylimidazolium hydrogen sulfate [16] and bmim chloride [17]. The interactions of alkylimidazolium cations with lignin can also lead to the formation of covalent bonds, for example, due to the base catalyzed nucleophilic addition of IL cation to carbonyl groups [18,19].

In a recent study [20] we proposed a scheme of wood fractionation with bmim acetate and its binary mixture with DMSO (80:20 *w*/*w*), which includes complete dissolution of sawdust at 120 °C with subsequent sedimentation of the polysaccharide and lignin fractions by acetone and acidic water, respectively. The yield of the obtained lignin fraction reached 55% of Klason’s lignin. The IL lignin samples were found to have a high content of nitrogen even after multiple rinses with water, which indicated the possibility of both a strong non-covalent binding of bmim cation (e.g., due to the formation of salts with acidic groups of lignin) and its covalent cross-linking with the biopolymer.

The current study is dedicated to a more detailed analysis of such IL lignin samples. In addition to bmim acetate, as an object we have also used bmim methylsulfate, which belongs to another class of ILs and also has a high dissolving power towards wood [17]. As the main methods of research two-dimensional NMR [21] and atmospheric pressure photoionization high-resolution mass spectrometry (APPI HRMS) [22,23] were chosen. Their combined use allows obtaining the most complete structural information for lignin characterization [24].

## 2. Results and Discussion

### 2.1. Molecular Weight Distributions of Ionic Liquid Lignins

**Figure 1:**
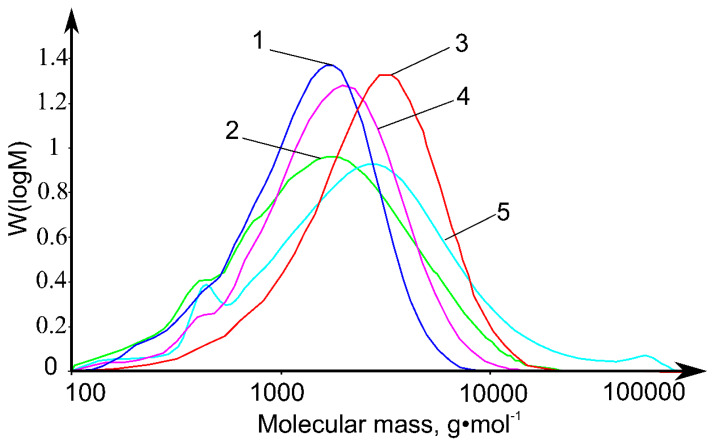
Molecular weight distributions of the lignin preparations isolated with [bmim]OAc (**1**), [bmim]OAc-DMSO (**2**), [bmim]MeSO_4_ (**3**), [bmim]MeSO_4_-DMSO (**4**), and dioxane lignin (**5**).

Molecular weight distributions of four studied IL lignin preparations (Figure 1) demonstrate unimodality with the molecular weights corresponding to the peak (M_p_) lying in a relatively narrow range of (2–4) × 10^3^ g·mol^−1^ and being close to this parameter for DL (3 × 10^3^ g·mol^−1^). The difference of IL lignins from dioxane lignin (DL) is in the lack of the highest molecular weight fractions (>2 × 10^4^ g·mol^−1^) and, accordingly, lower values of the number average (M_n_) and weight average (M_w_) molecular weights, as well as the polydispersity index (PDI) (Table 1). It can be caused by partial precipitation of the largest macromolecules together with a polysaccharide fraction characterized, as a result, by a high Kappa number [20].

It is remarkable that lignins obtained with bmim acetate are more destructed; M_n_ values for them are 1.5–2 times lower than for samples isolated with [bmim]MeSO_4_. At the same time, the addition of DMSO to [bmim]OAc does not significantly change the molecular weight characteristics of lignin, while in the case of [bmim]MeSO_4_ considerably reduces M_w_ and M_n_ preserving the PDI value. This difference may be due to the different acidity of [bmim]OAc and [bmim]MeSO_4_; the first IL is formed by a weak acid anion and belongs to the class of basic ILs, the second one contains a strong acid anion and promotes lignin condensation processes due to an increased acidity. The mechanism of DMSO effect on the molecular weight of lignin isolated with the use of [bmim]MeSO_4_ is not yet clear and may consist in prevention of side condensation processes by effective binding of residual water contained in the plant material, as well as solvation of protons and carbocations, which play an important role in the formation of C–C bonds between the structural links of lignin macromolecule.

### 2.2. Functional and Elemental Compositions of Ionic Liquid Lignins

The results of the functional analysis of the obtained IL lignin samples by NMR spectroscopy (^13^C and ^31^P) also reflect significant differences from DL preparation (Table 2).

A slightly increased content of carboxylic groups and a 20–25% decrease in the number of methoxyl groups are observed, which indicates that oxidation and demethoxylation side processes occur during fractionation. However, the most obvious changes refer to the content of hydroxyl and carbonyl groups. In the case of lignins isolated with the IL-DMSO system as well as [bmim]OAc, the contents of both aliphatic and phenolic hydroxyl groups were 1.5–2.5-fold lower compared to DL. For lignin isolated by [bmim]MeSO_4_, the difference is up to 6-fold in total hydroxyl content and almost 20-fold in aliphatic OH-groups content (Figure S1). These data are in contrast with a lower degree of IL lignin polymerization, which should correspond to a larger number of hydroxyl groups uninvolved in the formation of bonds between the structural units of lignin. This indicates the significant chemical transformations that lignin undergoes under the conditions of wood fractionation with IL. It includes partial acetylation of hydroxyl groups, which is evidenced by the presence of a significant number of acetyl groups in lignins isolated with [bmim]OAc (Table 2). In the case of [bmim]MeSO_4_ both methylation of hydroxyl groups and formation of keto-groups during cleavage of β-*O*-4 bonds is possible [12,13]. Since adding DMSO to [bmim]MeSO_4_ increases the number of free hydroxyl groups, it can be assumed that the solvent has a significant inhibitory effect on these processes.

The absence of aldehyde groups in lignins isolated with [bmim]OAc represents a particular interest. This may be related, for example, to the possibility of the addition of [bmim] cation to aldehyde groups which has been previously noted in the literature [18]. The process proceeds via deprotonation of bmim cation in the presence of bases, which can be both acetate ion and lower amine contaminants typical for IL. The elemental compositions of the obtained IL lignin samples (Table 3) demonstrate considerable nitrogen content which is an evidence for this suggestion. 

The presence of nitrogen can also be explained by the contamination with ionic liquid residues due to its incomplete removal during the washing of lignin with water. To discriminate covalently bound nitrogen and residues of free ionic liquid an additional purification of lignin samples by size-exclusion flash chromatography allowing rejection of low-molecular fraction was used. The obtained high-molecular weight fractions of IL lignins isolated with [bmim]OAc and its mixtures with DMSO (M_w_ ~4 × 10^3^ g·mol^−1^) contained 0.4% and 0.6% nitrogen, respectively. This corresponds to covalent binding of [bmim]^+^ cations in amounts up to 2% of lignin mass or up to three units per 100 guaiacylpropane structural fragments.

### 2.3. 2D NMR Spectra and Structural Characterization of Ionic Liquid Lignins

In order to provide more detailed structural characterization of the obtained IL lignin samples, two-dimensional NMR spectroscopy was used. HSQC spectra contain a large number of signals which overlap each other and complicate interpretation. They can be caused by the residual amounts of IL noted above, as well as the presence of decomposition products of both IL and lignin.

**Figure 2:**
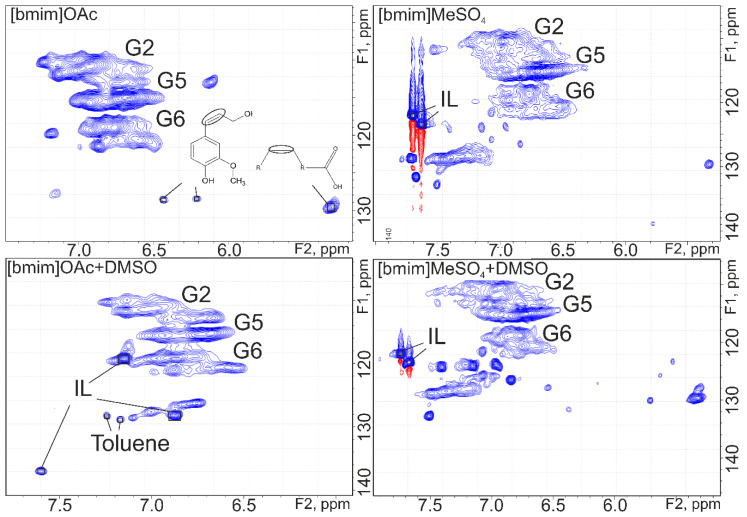
2D NMR spectra (HSQC) of IL lignins (aromatic structures region).

The area of aromatic structures (Figure 2) contains intense signals belonging to the atoms in the 2, 5 and 6 positions of the guaiacyl unit (G2, G5 and G6 respectively).

Intense signals related to the structure of unbound cation [bmim]^+^ were observed in the spectra of IL lignins obtained using [bmim]MeSO_4_ and its binary mixture with DMSO. It is confirmed by the presence of corresponding correlations in HMBC spectra. For lignin samples isolated from solutions in [bmim]OAc and [bmim]OAc-DMSO, these signals were not found, which may be due to the high basicity of IL and the formation of strong associates with lignin anions similar to the salt type. Signals at 32.2/3.93 ppm in the spectrum (in aliphatic region) of a sample obtained with [bmim]OAc-DMSO can be considered as an evidence for the presence of a covalent bond between the C2 atom of bmim and the α-atom of propane chain in the lignin structural unit. Similar signals occur in lignins isolated with [bmim]MeSO_4_ and [bmim]MeSO_4_-DMSO. However, due to the presence of IL contaminants, the position of the signal’s drifts to the area of smaller chemical shifts, preventing their reliable identification.

The recorded spectra also contain signals of 1-butyl-imidazole contaminants (a product of demethylation of IL) as well as toluene, which is formed, presumably, as a product of lignin degradation. The signals corresponding to the double bonds in coniferyl alcohol type structures were also clearly identified, confirming the process of destruction of lignin macromolecules.

**Figure 3:**
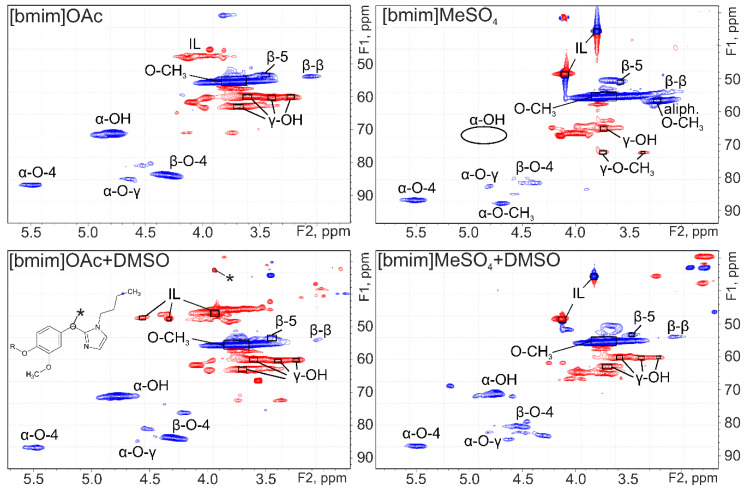
2D NMR spectra (HSQC) of IL lignins (aliphatic structures region).

Analysis of aliphatic oxygen-containing area in HSQC spectra (Figure 3) confirms the conclusions about possibility of chemical interaction of IL with lignin. Besides free IL signals, in the range δC/δH 50–44/4.3–3.7 ppm for lignins obtained with [bmim]OAc a number of signals belonging to the 1-butylimidazole residue covalently bound with lignin through the carbon atom located between the nitrogen atoms (based on HMBC spectra) are also observed. Guaiacyl units in the structure of the studied lignins are connected mainly by β-*O*-4, α-*O*-4/β-5, and α-*O*-γ/β-β bonds that form structural fragments of β-aryl ether, phenylcoumaran and pinoresinol, respectively. Quantitative estimation of the abundance of these bonds (Table 4) shows the similarity of all studied samples and approves a deeper degradation of ether bonds during the fractionation of wood with IL compared to the process of dioxane lignin extraction.

IL lignin obtained using [bmim]MeSO_4_ dramatically differs from other samples with abnormally low content of β-*O*-4 bonds, which are the weakest in lignin structure. This is additional evidence in favor of the hydrolytic effect of this ionic liquid on lignin and the assumption that side condensation processes occur in its medium with the formation of carbon–carbon bonds preventing the formation of low-molecular products of lignin destruction. In addition, an analysis of HSQC spectrum confirmed methylation reactions of aliphatic lignin hydroxyls in the presence of methyl sulfate anion. Addition of 20% DMSO eliminates these properties of [bmim]MeSO_4_ and provides obtaining lignins similar in their functional composition and structure to the studied samples isolated using IL with acetate anion.

The difference between lignins obtained using ILs and dioxane lignin (Figure S2) is also the absence of signals from vanillin, vanillic acid, hydroxymethylfurfural and monosaccharides. Thus, considering the values of molecular weights, polydispersity index and content of inter-unit bonds IL lignins are characterized by greater homogeneity of chemical composition.

### 2.4. High-Resolution Mass Spectrometry of Lignin Preparations

The data obtained by high-resolution mass spectrometry (Orbitrap) provided information on the chemical composition and variety of lignin oligomers in the studied samples. The obtained mass-spectra (Figure S3) contain up to 3000 peaks of deprotonated oligomer molecules in an *m*/*z* range of 250–1500 with relative intensity of more than 0.1%. Similar to the case of dioxane lignin [22], peaks are grouped into separate clusters corresponding to oligomers with different number of structural units (dimers-octamers). While the general appearance of mass spectra of lignins isolated with [bmim]OAc (Figure S3a,b) is similar to DL, significant differences are observed for lignins isolated with [bmim]MeSO_4_ (Figure S3c,d). They relate primarily to a narrower range of masses of detectable oligomers; signals of heptamers and higher molecular weight compounds are comparable to the noise level. This contradicts the data of size-exclusion chromatography and can be explained by the hypothesis of condensation processes with the formation of carbon–carbon bonds and the possibility of methylation of certain hydroxyl groups. The resulting structures may have lower ionization efficiency under APPI conditions.

**Figure 4:**
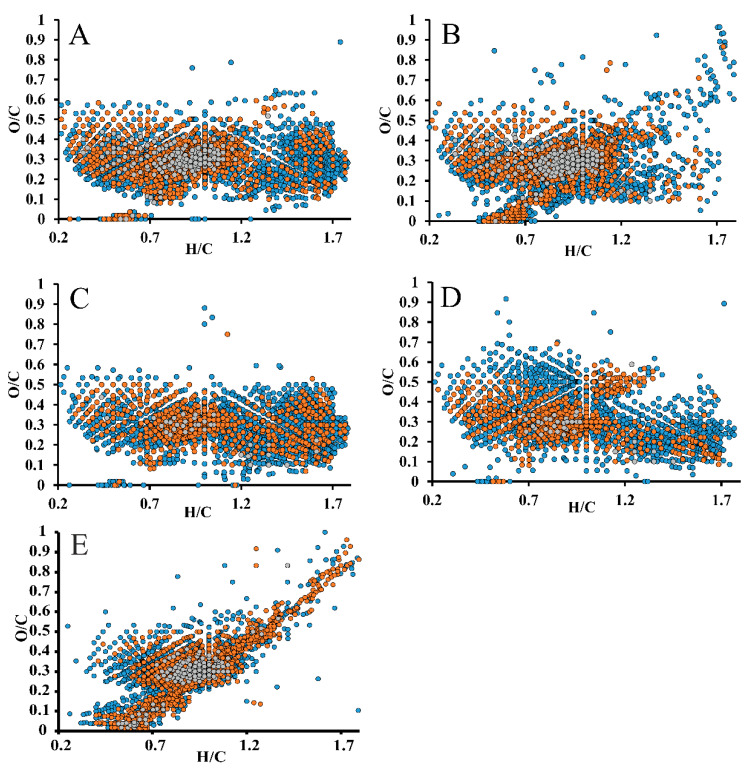
Van Krevelen plots for IL lignin preparations obtained using [bmim]OAc (**A**), [bmim]OAc-DMSO (**B**), [bmim]MeSO_4_ (**C**), [bmim]MeSO_4_-DMSO (**D**) and dioxane lignin (**E**). Dot colors designate the relative intensities of corresponding peaks in mass spectra: 0.1–1% (blue), 1–10% (brown), >10% (grey).

The most important data on the chemical composition of such complex objects as IL lignins can be obtained by analyzing their “images” in van Krevelen diagrams [25]. Compared to DL, the diagrams of the studied IL lignin preparations in O/C-H/C coordinates (Figure 4) are characterized by the absence of signals in the area of carbohydrate residues and lignin–carbohydrate complexes (H/C = 1.4–2.0, O/C = 0.6–1.0), as well as by the presence of multiple signals of low and medium intensity in the region of H/C = 1.3–1.8 and O/C = 0.1–0.5, corresponding to structures with a reduced degree of unsaturation (ring and double bond equivalent, RDB).

They can be associated with both the products of alkylation of lignin oligomers, and, partially, the products of decomposition of ILs and lignin. The most intense peaks in mass spectra related to this area correspond to compounds with elemental compositions C_28_H_44_O_3_, C_20_H_30_O_3_, C_20_H_34_O_9_, C_18_H_32_O_3_ etc. and RDB values of 3–7, which can contain no more than one benzene (or two furan) rings. Detailed study of their structures by tandem mass spectrometry is complicated because of impossibility to isolate the corresponding ions in the quadrupole mass filter due to the presence of a large number of isobaric compounds.

**Figure 5:**
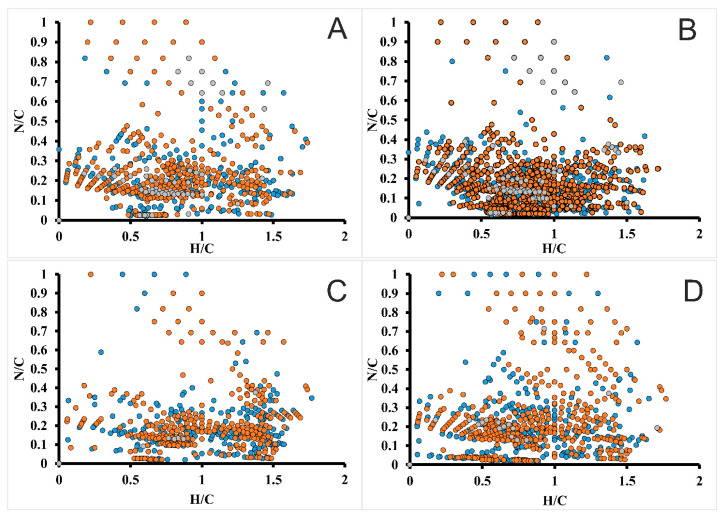
Van Krevelen plots for nitrogen-containing compounds in IL lignin preparations obtained using [bmim]OAc (**A**), [bmim]OAc-DMSO (**B**), [bmim]MeSO_4_ (**C**), [bmim]MeSO_4_-DMSO (**D**). Dot colors designate the relative intensities of corresponding peaks in mass spectra: 0.1–1% (blue), 1–10% (brown), >10% (grey).

The sample of DL does not comprise nitrogen-containing compounds in significant quantities, but in all four IL lignin preparations hundreds of components containing up to six nitrogen atoms and giving weak signals in mass spectra were detected. Elemental compositions of such structures (Figure 5) correspond mainly to the region of H/C = 0.5–1.5 and N/C = 0–0.3, shifted along the H/C axis by 0.1–0.2 units compared to CHO-class compounds.

They can be referred to products of binding of bmim cations to lignin oligomers, that leads to increase of H/C ratio. RDB values of detected nitrogen-containing compounds can be up to 30–35, which indirectly proves the presence of lignin fragments in their structure and corresponds to the data of NMR spectroscopy. It should be noted that the highest signal intensities and diversity of nitrogen-containing structures are typical for lignins isolated with the use of [bmim]OAc and its mixture with DMSO (Figure 5a,b), which also in a good agreement with the conclusions based on the study of functional and elemental composition of IL lignin samples.

## 3. Materials and Methods

### 3.1. Reagents and Materials

1-Butyl-3-methylimidazolium acetate and methyl sulfate (BASF quality, >95%) were purchased from Sigma-Aldrich (Steinheim, Germany). Before carrying out the experiments, ILs were dried over molecular sieve 4A (Neva Reaktiv, St. Peterburg, Russia), and the absence of residual moisture was controlled by infrared spectroscopy. For preparation of binary solvents dimethyl sulfoxide, “chem. pure” grade (Komponent-Reaktiv, Moscow, Russia), were used. Wood fractionation was carried out with the use of “chem. pure” grade acetone and hydrochloric acid (Komponent-Reaktiv, Moscow, Russia), deionized water obtained with Milli-Q system (Merk Millipore, Molsheim, France), and nitrogen (99.999%).

### 3.2. Lignin Preparations

*Picea abies* spruce wood (60–80 years of age) with lignin and cellulose content of 28% and 48%, respectively, has been used as a raw material for lignin preparations. The wood was prepared by cutting it into small pieces and grinding in a ball mill (Retsch, Haan, Germany) until the required particle size (<0.2 mm) was reached. In order to remove the extractives (resin acids, lipids, monomeric phenolic compounds) the obtained sawdust was extracted with acetone in the Soxhlet apparatus for 48 h, and then air-dried.

Four samples of IL lignins were obtained, with bmim acetate, bmim methyl sulfate and their binary mixtures with DMSO (80:20 *w*/*w*) respectively being used as solvents. Dissolution of the wood was carried out according to the previously described scheme [16] at 120 °C and the treatment duration 6 h. A 5 g load of sawdust was placed in a 250 mL three-necked flask containing 100 mL of IL or IL-DMSO mixtures and heated in an inert atmosphere (nitrogen flow) with constant stirring. As a result, full dissolution of wood was achieved. After treatment and cooling to the room temperature the obtained solution was poured to the flask containing 500 mL of acetone. Precipitated polysaccharide fraction was separated by filtration. The residual solution of lignin was concentrated on the rotary evaporator to a volume of 10–20 mL, mixed with excess of water and kept overnight at 4 °C under nitrogen atmosphere. Precipitated IL lignin was filtered and repeatedly washed with hot water, then dried under vacuum at 50 °C. The yields (recalculated to the oven-dried wood) of DL and IL lignins obtained with [bmim]OAc, [bmim]OAc-DMSO, [bmim]MeSO_4_ and [bmim]MeSO_4_-DMSO were 10.8%, 8.2%, 5.1%, 10.3% and 4.9 %, respectively.

Separation of IL lignin from low-molecular fraction containing IL residues was done by gel filtration. Sample (50 mg) was dissolved in 500 mL of DMSO and injected into a glass column (400 × 20 mm) filled with hydroxypropylated dextran gel Sephadex LH-20 (GE Healthcare, Uppsala, Sweden) and eluted with DMSO. The first fractions with Mn > 1000 Da were collected and combined for the further study. The obtained solution was evaporated to dryness under vacuum at 95 °C and subjected to elemental analysis.

In all experiments a sample of dioxane lignin (DL) extracted from the same wood with 0.1 M solution of HCl in aqueous dioxane (90%) by Pepper method [26] was used as a reference.

### 3.3. Analytical Procedures

Determination of the molecular weight distribution of the lignin preparations was carried out by an size-exclusion high-performance liquid chromatography using an LC-20 Prominence HPLC system (Shimadzu, Kyoto, Japan) consisting of a SIL-20A autosampler, an LC-20AD pump, a DGUA3 vacuum degasser, an STO-20A column thermostat and an SPD-20A spectrophotometric detector. Separation was performed at 50 °C on a Polargel-M column (Agilent, Santa Clara, CA, USA), 300 × 7.5 mm. Lithium bromide solution (0.0125 M) in DMF was used as a mobile phase with flow rate of 1 mL·min^−1^. Detection was performed at a wavelength of 275 nm. The system was calibrated with monodisperse polystyrene standards (PSS, Mainz, Germany) in a molecular weight range of 0.35–187 kDa.

The elemental composition was determined by catalytic combustion on a CHNS-analyzer EuroEA-3000 (EuroVector, Pavia, Italy).

NMR spectra were registered on an AVANCE III NMR spectrometer (Bruker, Ettlingen, Germany) with an operational frequency 600 MHz for protons. To determine the different types of hydroxyl groups by ^31^P-NMR the samples were dissolved in pyridine-*d*_5_ and derivatized by 2-chloro-4,4,5,5-tetramethyl-1,3,2-dioxophospholan (Sigma Aldrich, Darmstadt, Germany) [27]. To obtain ^13^C NMR spectra, about 30 mg of lignin was dissolved in 0.6 mL of DMSO-*d*_6_. Parameters of experiment: temperature of the sample—298 K, duration of the impulse—12 µs, registration time—0.9 s, delay between impulses—0.11 s, number of spectra accumulated—65000. To register 2D (^1^H-^13^C) HSQC (Heteronuclear Single Quantum Correlation) and HMBC (Heteronuclear Multiple Bond Correlation Spectroscopy) spectra the same lignin solution was used with addition of chromium tris-acetylacetotonate (~0.02 mol·L^−1^) as a relaxant. Experiment parameters: temperature—298 K, spectrum window width ~15 ppm for F2, and ~240 ppm for F1, with number of accumulations 1024 × 256, number of scans—32. The delay time between pulses (D1) was 2.0 s.

High resolution mass spectra were recorded according to a previously developed procedure [22,24]. The hybrid mass spectrometer Q Exactive Plus (Thermo Fisher Scientific, Waltham, MA, USA) with a quadrupole mass filter and mass analyzer based on an orbital ion trap at a resolution of 70,000 (FWHM, for *m/z* 200) and an Ion Max ion source with an atmospheric pressure photoionization system was used. A krypton gas discharge lamp with quantum energy of 10.0 (10.6) eV was used as a radiation source. Calibration of the mass scale was carried out in accordance with the manufacturer’s recommendation, using a Pierce mixture of standards (Thermo Fisher Scientific, USA). Flow injection (10 µL) of lignin solutions into the ion source with acetone flow (200 µL·min^−1^) created by chromatographic pump LC-30AD (Shimadzu, Kyoto, Japan) was used. Mass-spectra were recorded in the negative ionization mode in an *m/z* range of 300–2000 by averaging the results of at least 10 measurements and subtracting the background solvent signal. Optimal ion source parameters were used to ensure maximum intensity of mass spectra of the studied lignin preparation: drying gas pressure—20 psi, nebulizing and curtain gas flows—5 and 2 arbitrary units, respectively, desolvation line temperature—250 °C, vaporizer temperature—500 °C, radio frequency voltage on the S-lens—55 arbitrary units. Control of the mass spectrometer, data collection and processing were carried out using an Xcalibur software (Thermo Fisher Scientific, USA).

## 4. Conclusions

Ionic liquid lignins isolated by dissolution of wood in 1-butyl-3-methylimidazolium acetate and methyl sulfate and further sedimentation of polysaccharide and lignin fractions are characterized by the weight average molecular weight of (1.6–3.4) × 10^3^ g·mol^−1^ and differs from dioxane lignin by a higher degree of depolymerization and lower polydispersity. The wood fractionation process with the use of ionic liquids is accompanied by significant chemical transformations of lignin; a considerable decrease in the content of free phenolic hydroxyl groups due to alkylation and elimination (in the case of [bmim]OAc) of carbonyl groups, as well as destruction of β-*O*-4 bonds. Presence of nitrogen and sulfur (in the case of [bmim]MeSO_4_) in the elemental composition of IL lignins is an evidence in favor of chemical reactions with 1-butyl-3-methylimidazolium cations and methyl sulfate anions with covalent binding of ionic liquids (or products of their destruction) to lignin, which is confirmed by the data of 2D NMR spectroscopy and high resolution mass spectrometry. The addition of DMSO to IL (20%) has a significant effect on the properties of lignins obtained with [bmim]MeSO_4_ by preventing condensation processes and chemical modification of the biopolymer in a certain way. Further research should concentrate on a more detailed study of the chemical interactions of lignins with ionic liquids and the determination of the structures of the resulting products.

## Figures and Tables

**Table 1:** Molecular weight properties of isolated IL lignins and dioxane lignin.

Sample	M_w_, g·mol^−1^	M_n_, g·mol^−1^	PDI (M_w_/M_n_)
IL lignin ([bmim]MeSO_4_)	3.4	1.9	1.8
IL lignin ([bmim]MeSO_4_ + DMSO)	2.1	1.2	1.8
IL lignin ([bmim]OAc)	1.6	0.98	1.7
IL lignin ([bmim]Oac + DMSO)	2.1	0.98	2.1
DL	4.6	1.8	2.5

**Table 2:** Functional composition of isolated IL lignins and dioxane lignin determined by ^31^P and ^13^C-NMR spectroscopy.

Sample	Functional Groups and Their Content						
	as a % of Sample Weight			per 100 Aromatic Units			
	OH_total_	OH_phen._	OH_aliph._	COOH	OCH_3_	Acetyl	C=O_ald._
IL lignin ([bmim]MeSO_4_)	1.5	1.2	0.3	0.3	78.4	0	4.3
IL lignin ([bmim]MeSO_4_ + DMSO)	5.3	2.0	3.3	0.5	72.9	16.7	0
IL lignin ([bmim]OAc)	3.7	1.8	1.9	0.9	69.8	0	6.9
IL lignin ([bmim]OAc + DMSO)	4.6	2.1	2.6	0.3	69.3	15.3	0
DL	9.1	3.3	5.8	0.1	96.1	6.1	10.4

**Table 3:** Elemental composition of isolated IL lignins and dioxane lignin.

Sample	Elemental Composition, %				
	C	H	N	S	O
IL lignin ([bmim]MeSO_4_)	62.7	6.9	2.4	2.9	25.1
IL lignin ([bmim]MeSO_4_ + DMSO)	68.5	9.6	1.3	0.0	20.6
IL lignin ([bmim]OAc)	65.4	7.5	0.9	3.8	22.4
IL lignin ([bmim]OAc + DMSO)	66.6	8.9	4.5	0.0	20.0
DL	62.6	7.3	0.0	0.0	30.1

**Table 4:** Contents of the main types of inter-unit bonds in lignins (number per 100 aromatic units).

Sample	β-*O*-4	α-*O*-4/β-5	α-*O*-γ(γ-*O*-α)/β-β
[bmim]MeSO_4_	6.0	4.95	2.39
[bmim]OAc	20.8	4.68	3.38
[bmim]MeSO_4_-DMSO	15.1	6.28	2.10
[bmim]OAc-DMSO	15.0	4.25	2.18
DLS	21.4	7.37	3.53

## Supplementary Materials

The following are available online at https://www.mdpi.com/1420-3049/25/11/2479/s1, Figure S1: ^31^P-NMR spectra of IL lignins obtained with [bmim]OAc, [bmim]MeSO_4_ and their binary mixtures with DMSO after derivatization by 2-chloro-4,4,5,5-tetramethyl-1,3,2-dioxophospholan, Figure S2: 2D NMR spectrum (HSQC) of spruce dioxane lignin, Figure S3: High-resolution (Orbitrap) APPI mass spectra of IL lignins obtained with [bmim]OAc (A), [bmim]OAc-DMSO (B), [bmim]MeSO_4_ (C), [bmim]MeSO_4_-DMSO (D).

## References

[1] Faßbach T.A., Kirchmann R., Behr A., Vorholt A.J. (2017). Recycling of homogeneous catalysts in reactive ionic liquid–solvent-free aminofunctionalizations of alkenes. Green Chem..

[2] Konnerth H., Prechtl M.H.G. (2017). Selective Hydrogenation of N-Heterocyclic Compounds using Ru Nanocatalysts in Ionic Liquids. Green Chem..

[3] Bisht M., Mondal D., Pereira M.M., Freire M.G., Venkatesu P., Coutinho J.A.P. (2017). Long-term protein packaging in cholinium-based ionic liquids: Improved catalytic activity and enhanced stability of cytochrome c against multiple stresses. Green Chem..

[4] Kilpeläinen I., Xie H., King A., Granstrom M., Heikkinen S., Argyropoulos D.S. (2007). Dissolution of Wood in Ionic Liquids. J. Agric. Food Chem..

[5] Zhao H., Baker G.A., Wagle D.V., Ravula S., Zhang Q. (2016). Tuning task-specific ionic liquids for the extractive desulfurization of liquid fuel. ACS Sustain. Chem. Eng..

[6] Fox E.T., Weaver J.E.F., Henderson W.A. (2012). Tuning Binary Ionic Liquid Mixtures: Linking Alkyl Chain Length to Phase Behavior and Ionic Conductivity. J. Phys. Chem. C.

[7] Shu H., Xu Y. (2020). Tuning the strength of cation coordination interactions of dual functional ionic liquids for improving CO_2_ capture performance. Int. J. Greenh. Gas Control.

[8] Ladesov A.V., Kosyakov D.S., Bogolitsyn K.G., Gorbova N.S. (2015). Solvatochromic polarity parameters for binary mixtures of 1-butyl-3-methylimidazolium acetate with water, methanol, and dimethylsulfoxide. Russ. J. Phys. Chem. A.

[9] Lan W., Liu C.-F., Sun R.-C. (2011). Fractionation of Bagasse into Cellulose, Hemicelluloses, and Lignin with Ionic Liquid Treatment Followed by Alkaline Extraction. J. Agric. Food Chem..

[10] Liu C., Li Y., Hou Y. (2019). Behavior of oxygen-containing groups in grass lignin during dissolution in basic ionic liquids. Cellulose.

[11] Achinivu E.C. (2018). Protic ionic liquids for lignin extraction—A lignin characterization study. Int. J. Mol. Sci..

[12] Moghaddam L., Zhang Z., Wellard R.M., Bartley J.P., O’Hara I.M., Doherty W.O.S. (2014). Characterisation of lignins isolated from sugarcane bagasse pretreated with acidified ethylene glycol and ionic liquids. Biomass Bioenergy.

[13] Wen J.-L., Sun S.-L., Xue B.-L., Sun R.-C. (2013). Quantitative Structures and Thermal Properties of Birch Lignins after Ionic Liquid Pretreatment. J. Agric. Food Chem..

[14] Pinkert A., Goeke D.F., Marsh K.N., Pang S. (2011). Extracting wood lignin without dissolving or degrading cellulose: Investigations on the use of food additive-derived ionic liquids. Green Chem..

[15] Brandt A., Chen L., van Dongen B.E., Welton T., Hallett J.P. (2015). Structural changes in lignins isolated using an acidic ionic liquid water mixture. Green Chem..

[16] Qu Y., Luo H., Li H., Xu J. (2015). Comparison on structural modification of industrial lignin by wet ball milling and ionic liquid pretreatment. Biotechnol. Rep..

[17] Sowmiah S., Srinivasadesikan V., Tseng M.-C., Chu Y.-H. (2009). On the Chemical Stabilities of Ionic Liquids. Molecules.

[18] Chiarotto I., Feroci M., Inesi A. (2017). First direct evidence of N-heterocyclic carbene in BMIm acetate ionic liquids. An electrochemical and chemical study on the role of temperature. New J. Chem..

[19] Ladesov A.V., Belesov A.V., Kuznetsova M.V., Pochtovalova A.S., Malkov A.V., Shestakov S.L., Kosyakov D.S. (2018). Fractionation of Wood with Binary Solvent 1-Butyl-3-methylimidazolium Acetate + Dimethyl Sulfoxide. Russ. J. Appl. Chem..

[20] Brandt A., Hallett J.P., Leak D.J., Murphy R.J., Welton T. (2010). The effect of the ionic liquid anion in the pretreatment of pine wood chips. Green Chem..

[21] Wen J.-L., Sun S.-L., Xue B.-L., Sun R.-C. (2013). Recent Advances in Characterization of Lignin Polymer by Solution-State Nuclear Magnetic Resonance (NMR) Methodology. Materials.

[22] Kosyakov D.S., Ul’yanovskii N.V., Anikeenko E.A., Gorbova N.S. (2016). Negative ion mode atmospheric pressure ionization methods in lignin mass spectrometry: A comparative study. Rapid Commun. Mass Spectrom..

[23] Pikovskoi I.I., Kosyakov D.S., Shavrina I.S., Ul’yanovskii N.V. (2019). Study of Nettle (Urtica dióica) Lignin by Atmospheric Pressure Photoionization Orbitrap Mass Spectrometry. J. Anal. Chem..

[24] Pikovskoi I.I., Kosyakov D.S., Faleva A.V., Shavrina I.S., Kozhevnikov A.Y., Ul’yanovskii N.V. (2020). Study of the Carex Lignin by high-resolution mass spectrometry and nuclear magnetic resonance spectroscopy. Russ. Chem. Bull..

[25] Van Krevelen D. (1950). Graphical statistical method for the study of structure and reaction processes of coal. Fuel.

[26] Pepper J.M., Baylis P.E., Adler E. (1959). The isolation and properties of lignin obtained by the acidolysis of spruce and aspen woods in dioxane-water. Can. J. Chem..

[27] Granata A., Argyropoulos D.S. (1995). 2-Chloro-4,4,5,5-tetramethyl-1,3,2-dioxaphospholane, a reagent for the accurate determination of the uncondensed and condensed phenolic moieties in lignins. J. Agric. Food Chem..

