# Supplementary material for: Characterization of Ionic Liquid Lignins Isolated from Spruce Wood with 1-Butyl-3-methylimidazolium Acetate and Methyl Sulfate and Their Binary Mixtures with DMSO
Source: Molecules. 2020 May 27;25(11):2479. doi: 10.3390/molecules25112479 (PMC7321317; doi:10.3390/molecules25112479)
Supplement: Supplementary file 1 [file molecules-25-02479-s001.pdf]

molecules

Supplementary Materials

Characterization of Ionic Liquid Lignins Isolated from Spruce Wood with 1-Butyl-3-methylimidazolium Acetate and Methyl Sulfate and their Binary Mixtures with DMSO

Artyom V. Belesov, Anton V. Ladesov, Ilya I. Pikovskoi, Anna V. Faleva and Dmitry S. Kosyakov*

Core Facility Center 'Arktika', Northern (Arctic) Federal University, Arkhangelsk, 163002 and Russia

*Correspondance: d.kosyakov@narfu.ru

Abstract: Ionic liquids (ILs) based on 1-butyl-3-methylimidazole (bmim) have proved to be promising solvents for fractionation of plant biomass with the production of cellulose and lignin. This study deals with the characterization of lignins isolated from coniferous (spruce) wood using [bmim]OAc and [bmim]$MeSO_4$ ionic liquids and their binary mixtures with DMSO (80:20). Molecular weight distributions, functional composition and structural features of IL lignins were studied by size-exclusion chromatography, NMR spectroscopy (1D and 2D) and atmospheric pressure photoionization high-resolution mass spectrometry. It was shown that the interaction of ILs with lignin leads to significant chemical changes in the biopolymer - a decrease in the degree of polymerization and the content of free phenolic hydroxyl groups due to alkylation, the disappearance (in the case of [bmim]OAc) of carbonyl groups, a significant destruction of β-O-4 bonds. The chemical reactions between lignin and 1-butyl-3-methylidazolium cation with covalent binding of ionic liquids or products of their decomposition is evidenced by the presence of a large number of nitrogen-containing oligomers in IL lignins.

Keywords: lignin; ionic liquids; 1-butyl-3-methylimidazolium; wood fractionation

---

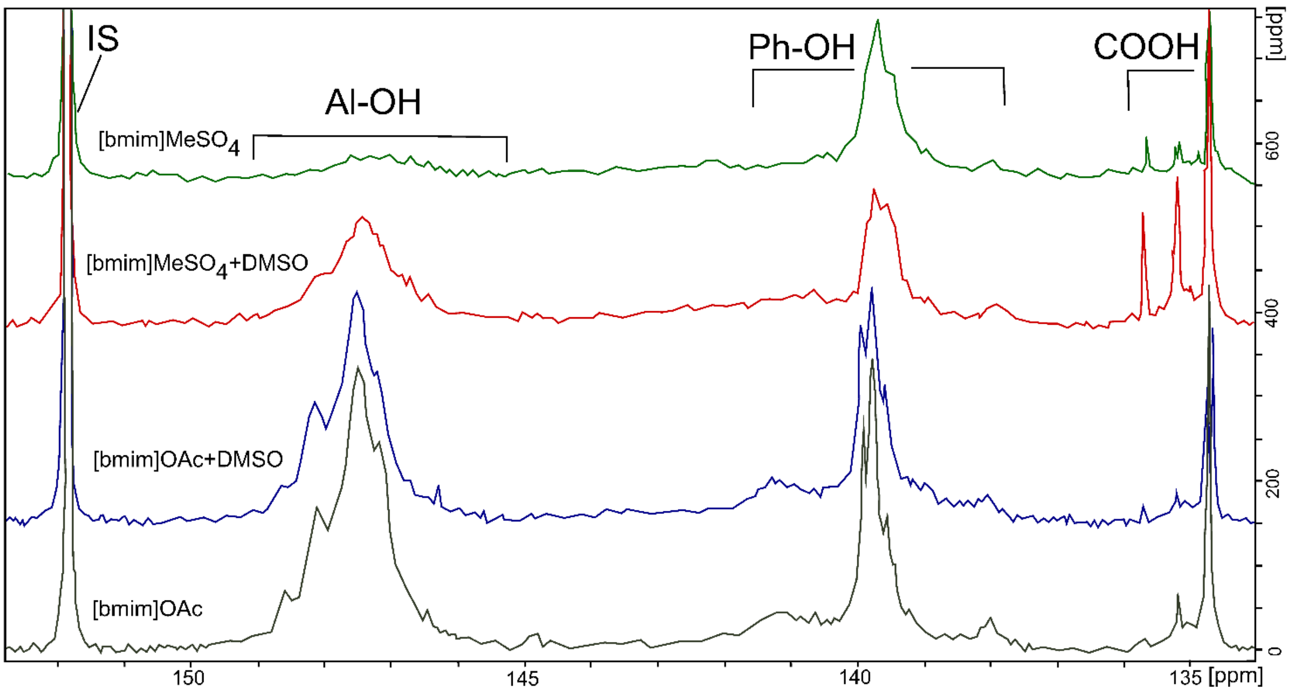

Figure S1. ^{31}P NMR spectra of IL lignins obtained with [bmim]OAc, [bmim]MeSO4 and their binary mixtures with DMSO after derivatization by 2-chloro-4,4,5,5-tetramethyl-1,3,2-dioxophospholan

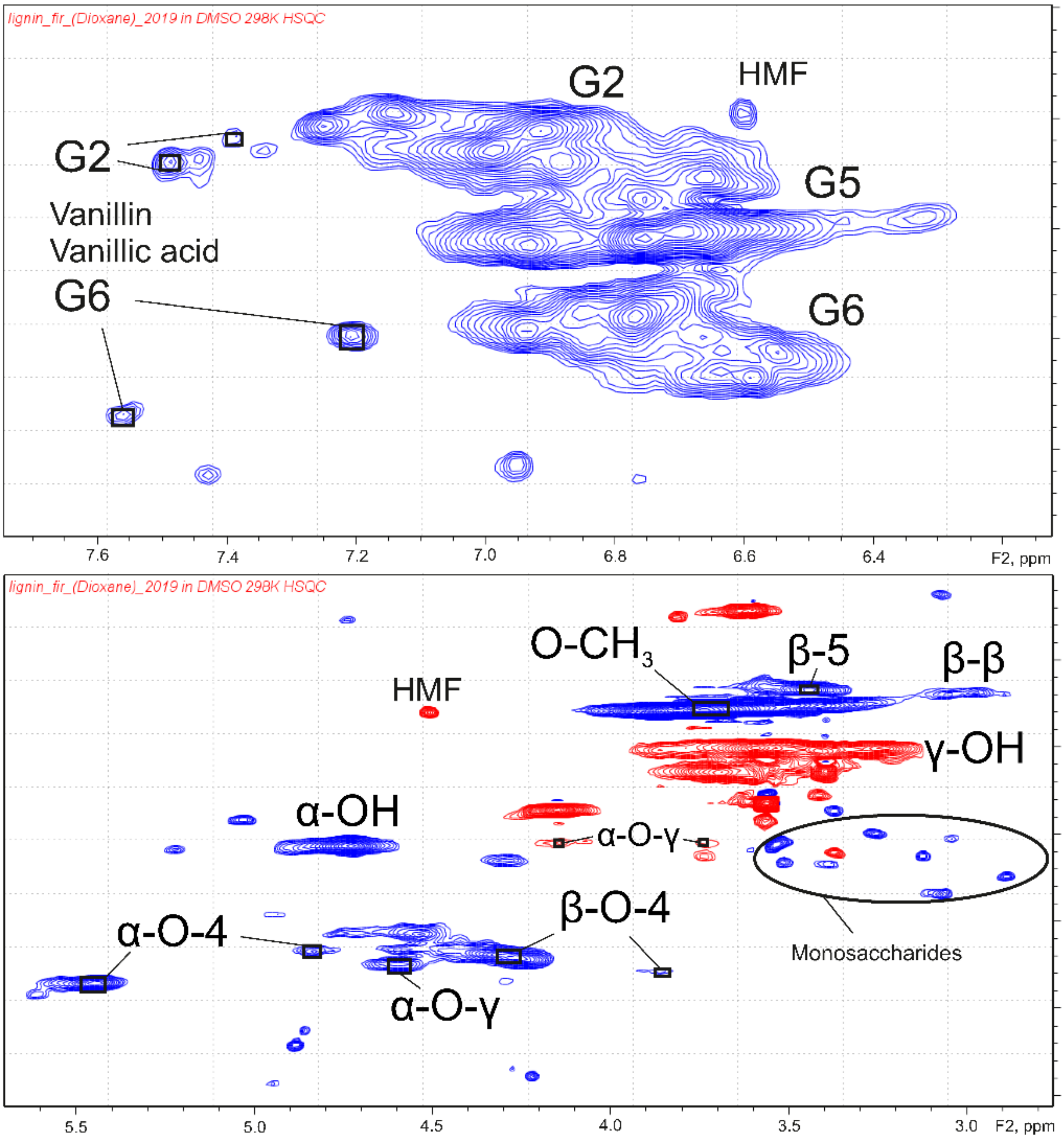

Figure S2. 2D NMR spectrum (HSQC) of spruce dioxane lignin.

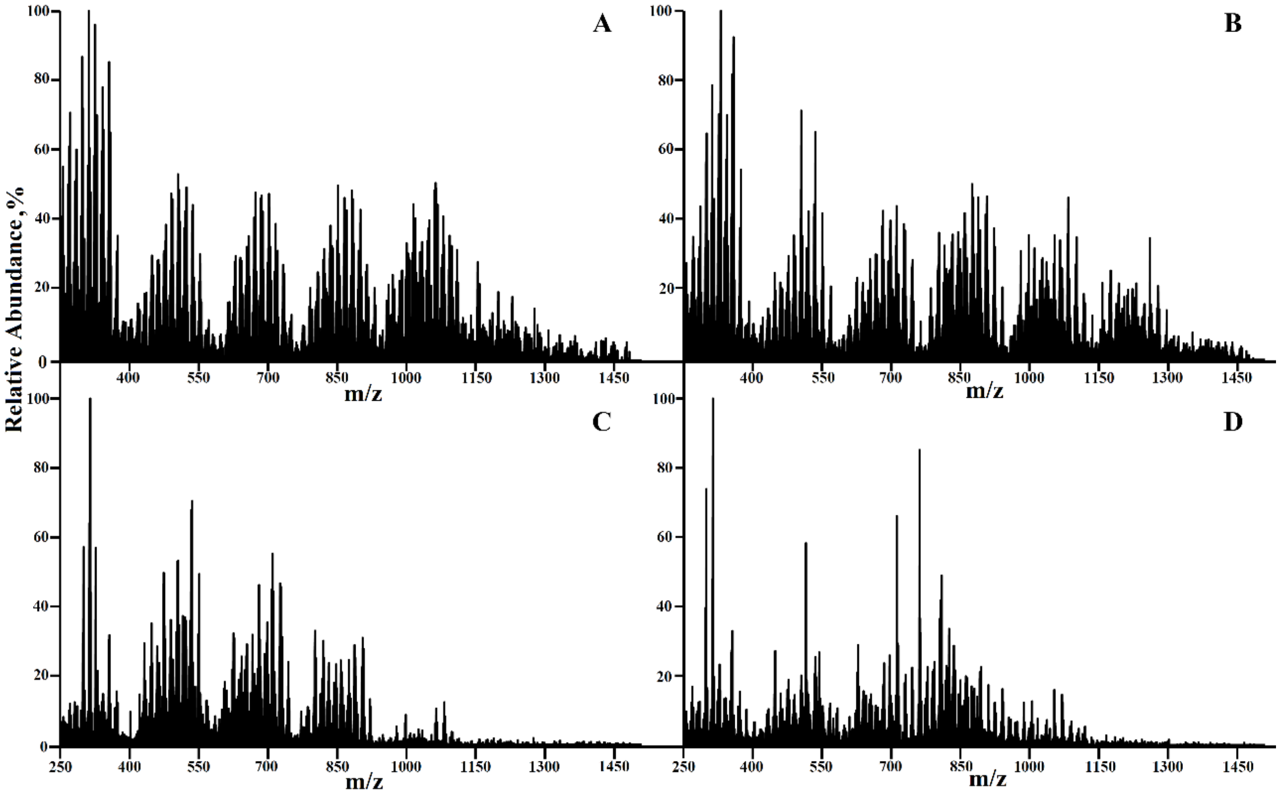

Figure S3. High-resolution (Orbitrap) APPI mass spectra of IL lignins obtained with [bmim]OAc (A), [bmim]OAc-DMSO (B), [bmim]$MeSO_4$ (C), [bmim]$MeSO_4$-DMSO (D)
